# A Practical Approach to the Diagnosis and Management of Hair Loss in Children and Adolescents

**DOI:** 10.3389/fmed.2017.00112

**Published:** 2017-07-24

**Authors:** Liwen Xu, Kevin X. Liu, Maryanne M. Senna

**Affiliations:** ^1^Harvard Medical School, Boston, MA, United States; ^2^Department of Dermatology, Massachusetts General Hospital, Boston, MA, United States

**Keywords:** alopecia, hair diseases, pediatrics, hair loss treatment, alopecia areata, tinea capitis, aplasia cutis congenita

## Abstract

Hair loss or alopecia is a common and distressing clinical complaint in the primary care setting and can arise from heterogeneous etiologies. In the pediatric population, hair loss often presents with patterns that are different from that of their adult counterparts. Given the psychosocial complications that may arise from pediatric alopecia, prompt diagnosis and management is particularly important. Common causes of alopecia in children and adolescents include alopecia areata, tinea capitis, androgenetic alopecia, traction alopecia, trichotillomania, hair cycle disturbances, and congenital alopecia conditions. Diagnostic tools for hair loss in children include a detailed history, physical examination with a focused evaluation of the child’s hair and scalp, fungal screens, hair pull and tug test, and if possible, light microscopy and/or trichoscopy. Management of alopecia requires a holistic approach including psychosocial support because treatments are only available for some hair loss conditions, and even the available treatments are not always effective. This review outlines the clinical presentations, presents a diagnostic algorithm, and discusses management of these various hair loss disorders.

## Introduction

Hair loss in children encompasses a spectrum of conditions congenital and acquired, originating from hair shaft, follicular, or infectious causes. A congenital hair abnormality may be an isolated finding in an otherwise healthy child or a feature suggestive of a multisystem syndrome. Both congenital and acquired alopecia may be irreversible, resulting from destruction of hair follicles and replacement by fibrous scar tissue. Disease processes that lead to follicular loss are known as scarring alopecia, whereas follicles are generally preserved in non-scarring alopecia (1).

A basic understanding of hair biology enables consideration of normal versus abnormal hair loss in pediatric patients. Hair consists of the proteinaceous shaft and the root, anchored in the follicle, an involution of the epidermis. Hair follicles contain rapidly dividing cells, but the only visible portions are the follicular ostia, through which hair fibers emerge. Humans are born with a population of approximately five million preformed follicles. Follicles can produce lanugo, vellus, or terminal hairs. Newborns are covered in soft lanugo hair, which is replaced by downy, fine vellus hair after 3–4 months. At puberty, androgens transform most vellus hair into thicker, more pigmented terminal hair. Terminal hairs undergo cyclical growth with up to 90% of follicles in active growth (anagen phase); 1–3% in a brief involutionary transition (catagen phase); and 5–10% in dormancy (telogen phase). Hair is shed after 2–3 months in telogen phase, after which anagen begins again, and the cycle repeats. Daily loss of 50–150 telogen hairs is normal, but the number of hairs shed is roughly equal to the number of follicles entering anagen (2, 3).

Clinical presentation of pediatric alopecia ranges from subtle to disfiguring. A thorough history provides context as to whether hair was present at birth, or establishes acuity if hair was later lost. Other relevant details include diet and nutrition, environmental exposure to toxins, past medical history, and family history, with special attention to atopic, autoimmune, and endocrine diseases. Physical examination should focus on scalp health, followed by a survey of lashes, dentition, nails, and skin. Clinical findings, such as short stature, dysmorphic features, and vision or hearing impairment, could indicate an underlying syndrome.

**Figure 1:**
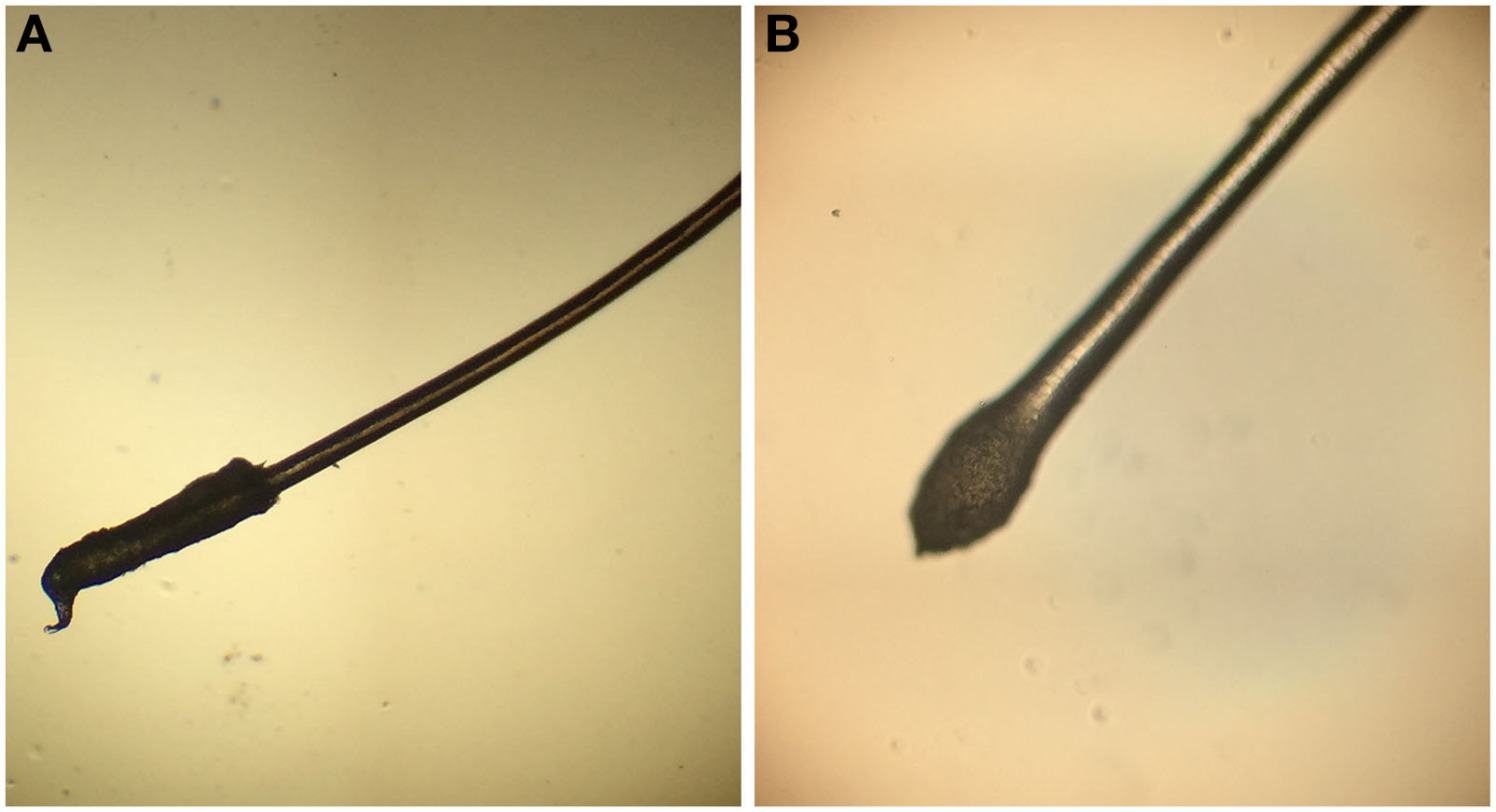
**(A)** The anagen hair root is covered by a long sheath. **(B)** The telogen hair root is club shaped and without a sheath.

Scalp examination reveals distribution of hair loss (diffuse, patterned, and focal) and the areas most affected (i.e., vertex, occiput, bitemporal, periphery). Trichoscopy is the application of a dermatoscope to directly visualize the scalp and hair, facilitating closer looks at regions of active disease. Trichoscopy is increasingly utilized in diagnosis of alopecias as trichoscopic features of common hair and scalp diseases are being recognized and organized (4–7). Notably, loss of follicular ostia identifies scarring alopecias, and preserved ostia indicate likely non-scarring alopecias. Trichoscopy also allows for identification of interfollicular stigmata, such as erythema, edema, pustules, and altered skin pigmentation or atrophy. As a non-invasive tool, trichoscopy is practical for use in children for diagnostic purposes and evaluating responses to therapeutic interventions (8). Hair quality can be evaluated with simple clinical maneuvers including the tug and pull tests. The tug test involves grasping hair between the thumb and forefinger near its root and tugging with the other hand on the same strand at its distal part and is positive if the hair fractures, indicating hair shaft fragility (9). To perform the hair pull test, gently pull a bundle of 20–60 hairs between the thumb and forefinger from multiple locations. The pull test is positive if greater than 10% of hairs are released; the test is uninfluenced by time since last hair wash (10, 11). In general, telogen hair comes out easily whereas anagen hair remains rooted in place. However, it is always important to evaluate roots of pulled hair under a microscope to definitively distinguish telogen from anagen hairs (Figure 1). Moreover, microscopy can exclude the presence of anagen hairs with thickened root sheaths, which would strongly suggest scarring alopecia even if the pull test does not reveal increased hair loss (12).

**Figure 2:**
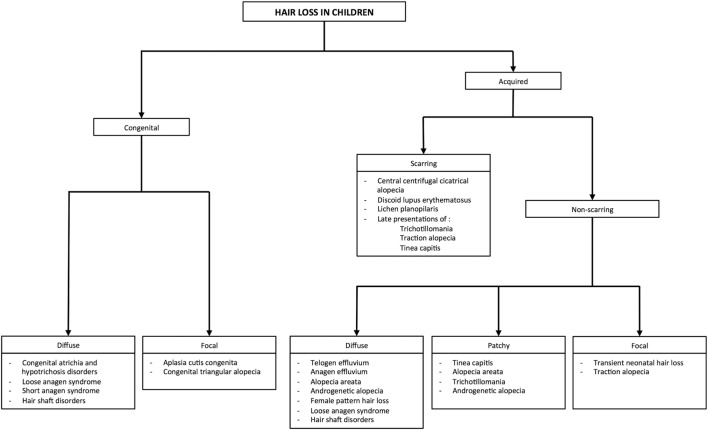
Approach to common etiologies of hair loss in children and adolescents.

The majority of pediatric alopecia is due to an acquired cause, most commonly tinea capitis, alopecia areata (AA), telogen effluvium, and traumatic hair loss, including trichotillomania and traction alopecia. If alopecia is present at birth and a congenital cause is suspected, aplasia cutis congenita (ACC) should be considered in the differential diagnosis. None of the above disease processes target and damage the follicle specifically; hence, all are non-scarring etiologies. However, tinea capitis and traumatic hair loss can be scarring from longstanding inflammation if left untreated. To aide the general pediatrician with distinguishing between various causes of alopecia, we include a diagnostic pathway that encompasses these and other congenital and acquired causes of hair loss in children (Figure 2; Table 1).

**Table 1:** Important clinical findings of alopecias to aid in diagnosis.

Type of hair loss	Loss of follicular ostia	Pattern	Scale	Erythema	Trichoscopy	Trichogram	Classic findings
Alopecia areata	No	Patchy, diffuse, or complete hair loss	No	None to mild	Yellow dots, exclamation mark hairs	Tapered hair	“Exclamation point” hairs, ± nail pitting, ± family history of AA

Anagen effluvium	No	Diffuse	No	No			Occurs days or weeks after inciting event

Androgenetic alopecia	No	Patchy, usually vertex or temporoparietal regions	No	No	Thin and vellus hairs, hair shaft thickness diversity, perifollicular pigmentation, yellow dots	Diversity of hair shaft thickness	Vellus hairs within patches, ↑ androgen levels, ± family history

Aplasia cutis congenita	No	Focal	No	No	Telangiectasia, radially oriented hair follicles with visible bulbs under translucent epidermis		Absent, thin or ulcerated overlying skin, surrounded by ring of dark, coarse hair “collar”

Central centrifugal cicatricial alopecia	Yes	Patchy, starting at vertex	Yes	Yes	Peripilar gray/white halo, disrupted pigmented network		Hair loss gradually spreads centrifugally, ± family history

Congenital atrichia and hypotrichosis	No, follicular agenesis	Diffuse	No	No			Complete hair loss by >2 years, isolated finding or feature of syndrome

Congenital triangular alopecia	No	Focal, often unilateral, temporal region	No	No	Vellus hairs, miniaturized terminal hair follicles, hair length diversity		Vellus hairs within patches, ± peripheral terminal hairs, does not improve with age

Discoid lupus erythematosus	Yes	Patchy, diffuse	Yes	Yes	Yellow dots with radial, thick, arborizing vessels		Confluent erythema and scale, follicular plugging

Female pattern hair loss	No	Diffuse, “Christmas tree pattern” on top of scalp	No	No	Yellow dots, single-hair pilosebaceous units, perifollicular hyperpigmentation		Often little to no evidence of androgen excess

Lichen planopilaris	Yes	Patchy, diffuse	Yes	Yes			Perifollicular erythema and scales, scalp atrophy

Loose anagen syndrome	No	Diffuse	No	No	Rectangular black granular structures, solitary yellow dots, and >90% of follicular units with single hairs	Ruffled cuticle, absent root sheaths	Tend to occur in female infants, improves with age

Short anagen syndrome	No	Diffuse	No	No		Telogen hair with tipped point	Normal hair density, but with very short hairs

Structural hair disorders	No	Diffuse	No	No	Features specific to different hair shaft abnormalities (107)	Fragmented hair shaft; increase hair breakage	Variation in hair texture and appearance, (+) hair tug test

Telogen effluvium	No	Diffuse	No	No		Increased percentage of clubbed hairs	Occurs approximately 3 months after inciting event or illness, (+) hair pull test if active disease

Tinea capitis	Yes and no	Patchy	Yes	Yes			Black dot or “comma” hair, posterior cervical LAD, (+) KOH test

Traction alopecia	Yes and no	Patchy, usually temporal or frontomarginal regions	Yes	Yes			Fringe sign, history of grooming practices causing excessive scalp tension, ± folliculitis, (−) hair pull test

Transient neonatal hair loss	No	Focal	No	No			Improves with age

Trichotillomania	No	Patchy with irregular borders	None to mild	None to mild	Broken hairs, “V-sign,” tulip hairs		Various stages of hair regrowth, ± loss of eyebrow or lashes, (−) hair pull test, personal or family psychiatric history

*LAD, lymphadenopathy; KOH, potassium hydroxide*.

## Tinea Capitis

**Figure 3:**
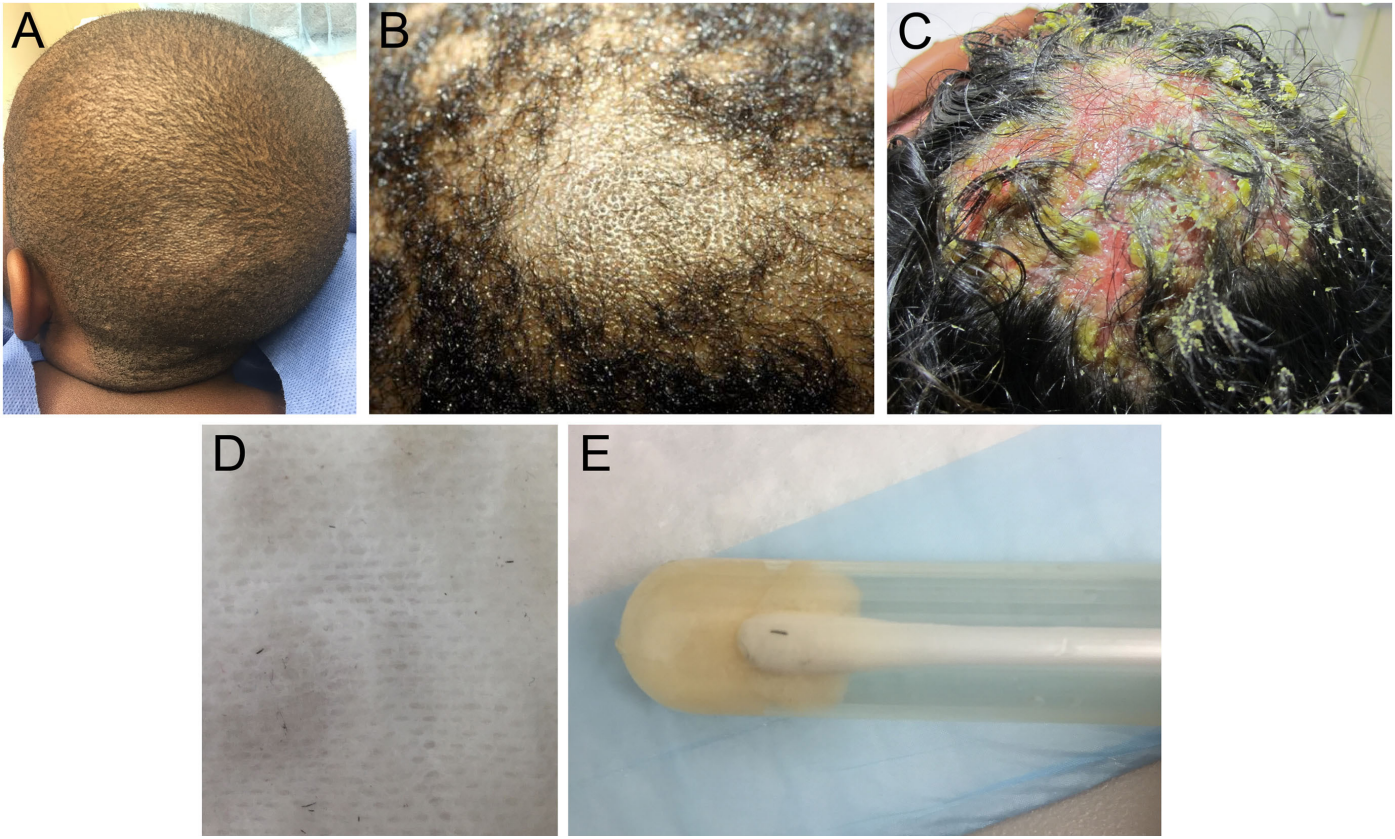
**(A)** Patchy hair loss and broken hairs in a child with tinea capitis infection. **(B)** A boggy mass representing a kerion. **(C)** A large pustular kerion. **(D)** Black dots are remnants of broken hair, which have been removed on a gauze with gentle rubbing. **(E)** Close-up of broken hairs in culture tube.

Tinea capitis (Figure 3) is a common dermatophyte infection that primarily affects children, including infants. In the United States, *Trichophyton tonsurans* and *Microsporum canis* are the most common causative organisms. *Trichophyton* can spread human-to-human, whereas *Microsporum* can be acquired from pets (13, 14). After direct inoculation, the dermatophytes invade the hair follicle, resulting in acute patchy hair loss with various degrees of erythema, pruritus, and scaling (15). Kerions are boggy, sometimes purulent plaques that indicate presence of increased local inflammation (Figures 3B,C). Rare in infancy, kerions can present in children between ages 5 and 10 (16).

Differential diagnosis of tinea capitis includes AA and ACC. The presence of black dot hairs (broken hairs within the lesion), significant erythema and scaling, and posterior cervical lymphadenopathy all favor tinea capitis. Both a swab culture of the affected skin and a hair from the affected area should be submitted for fungal culture (Figures 3D,E). Fungal potassium hydroxide preparations rapidly identify dermatophytes, and dermatophyte culture will confirm the diagnosis. Notably, sampling kerions can yield false-negative cultures because kerions mostly reflect an intense inflammatory response (17). Patients can be successfully treated with oral antifungal medications (e.g., terbinafine and griseofulvin) plus antifungal shampoos such as ketoconazole 2% shampoo to prevent shedding. Many children under 1 year can be adequately treated with topical azoles alone. Kerions can be managed with gentle soaks or keratolytic emollients (18, 19). Since dermatophyte infections can be spread to close contacts, sharing of combs, hairbrushes, and hats should be avoided. In some cases, treatment of close contacts might include preventative use of ketoconazole 2% shampoo for hair washing.

## Alopecia Areata

Alopecia areata is a common form of autoimmune hair loss with a lifetime prevalence of approximately 2%, occurring at any age (20). Approximately 20% of patients are under 16 and 1–2% present before age 2 (21, 22). The lifetime risk for AA is increased in individuals with personal or family history of other autoimmune disorders such as vitiligo or thyroid disease (23). The genetic basis for AA is still under investigation; however, genes that control T-cell proliferation and activation, including CTLA4, interleukins (IL2/IL21), human leukocyte antigens, and natural killer cell-activating ligands, have been implicated in AA pathogenesis (24).

**Figure 4:**
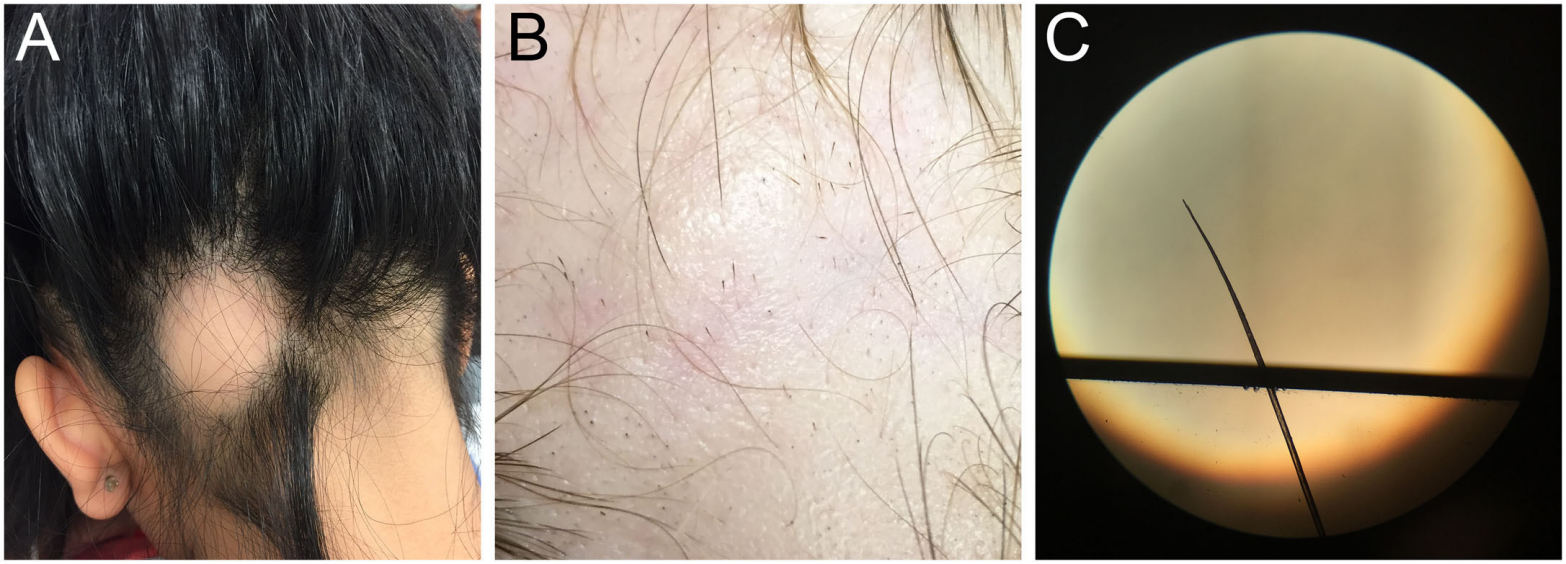
**(A)** Single, well-defined patch of hair loss characteristic of alopecia areata (AA). **(B)** Pathognomonic exclamation point hairs in AA. **(C)** Light microscopic appearance of a tapered hair removed from the scalp of patient with rapidly progressive AA.

The clinical course of AA is typified by patchy hair loss on the scalp (Figure 4A), though some individuals progress to have hair loss of the entire scalp [alopecia totalis (AT)] or the entire body [alopecia universalis (AU)]. It has been estimated that approximately 5% of patients with AA will develop AT and 1% will go on to develop AU. Although patches of hair loss can develop randomly over the scalp, there are patterns of hair loss that can occur as well, such as the band-like pattern of hair loss on the lower occiput scalp from ear to ear (ophiasis type), or as vertex hair loss with occipital and temporal sparing (sisaipho type) (25). Clinical diagnosis of early disease can often be made based on observing non-scarring, well-demarcated areas of complete hair loss, exclamation mark hairs observed at the lesion peripheries (Figure 4B), and positive hair pull with microscopic evidence of tapered hair (Figure 4C). Nail changes, including trachyonychia (rough vertically arranged linear ridges in the nail plate) and pitting (pinpoint dotted depressions in the nail plate), may be associated with AA. Clinical features that suggest a more chronic, relapsing course include a family history of AA, evidence of nail changes, presentation of areata during childhood, duration of current hair loss episode greater than 1 year, patient history of atopy, and more severe involvement (AT or AU). Though rarely necessary, scalp biopsies demonstrate the histological hallmark of dense lymphocyte infiltration of anagen hair bulbs and dermal papillae.

No consistently effective treatment exists for AA, with even fewer therapeutic options for children. Patients with a few patches of alopecia can be managed by watchful waiting as spontaneous regrowth can occur within 6–12 months in up to 50%. Children under 10 years often respond well to topical steroids (15). The side effect profile is considered benign, although development of rare systemic effect must be monitored. As hair regrows, topical steroids should be cautiously continued while watching for dermal atrophy. For children over 10 years with limited areas of hair loss, intralesional steroid injections may be trialed as tolerated. In our experience, treatments can be performed rather painlessly with the use of a topical anesthetic and vibrational device during injections. Systemic corticosteroids are reserved for AA refractory to other local treatment (26). Non-steroidal treatments include phototherapy, topical minoxidil, topical immunotherapy, such as squaric acid dibutyl ester and diphencyprone, and immunomodulators. Squaric acid dibutyl ester can increase hair growth particularly in children with extensive involvement, although side effects include itchy dermatitis, blisters, cervical lymphadenopathy, and hyperpigmentation. Children with AT or AU are often poor responders (27, 28). Diphencyprone has some efficacy in children with extensive involvement and the regrowth may be maintained in some patients even after discontinuation of diphencyprone (29). Systemic treatment with oral tofacitinib, a Janus kinase 1/3 inhibitor, has recently been reported as a successful treatment in pediatric patients with extensive AA. In a study of 13 patients’ ages 12–17 years, 9 patients (70%) had clinically significant hair regrowth after approximately 6 months of treatment. Although long-term side effects including potential for malignancy are unknown and require discussion with patient and family, overall initial adverse events appear to be mild and largely limited to upper respiratory infections, headache, and transient mild elevations in liver transaminase levels that returned to normal despite continued tofacitinib treatment (30). However, AA in its widespread forms remains hardest to treat, and no treatment appears to prevent relapse.

## Hair Cycle Disturbances

### Telogen Effluvium

Telogen effluvium is a common non-scarring hair cycle disorder in which an abnormal amount of hairs transitions from anagen phase to telogen phase, resulting in increased diffuse shedding of hair (19, 31). This growth cycle perturbation is usually due to medications, illness, surgery, metabolic disturbances, nutritional deficiencies, trauma, immunizations, and the postpartum state. However, no trigger is identified in one-third of cases (19, 31–34). Moreover, it is not uncommon for telogen effluvium to be superimposed on other forms of alopecia if patients experience an inciting event. Important differential diagnoses to consider include androgenetic alopecia (AGA), female pattern hair loss (FPHL), and diffuse AA (35, 36). Acute telogen effluvium occurs 3–4 months after an inciting event, lasts approximately 3 months, and spontaneously resolves, while chronic telogen effluvium usually lasts longer than 6 months. The hair pull test roughly estimates the percentage of telogen hairs. A positive hair pull test, particularly at the vertex and the scalp margins, is highly suggestive of telogen effluvium (19, 31, 33).

Patients can present after the phase of acute shedding has subsided. In these cases, patients report a period of increased shedding, while on exam, clinicians observe evidence of short hairs regrowing along the temporal and frontal hairline. A complete blood count and metabolic panel, with additional tests for metal poisoning and nutrition deficiencies, can help identify causes of telogen effluvium (19, 37). Acute telogen effluvium is completely reversible with removal of stressors and treatment of underlying disorders, if present. Prognosis is generally good with most patients regrowing hair within the year, though at a slow rate of approximately 1 cm/month (19). Treatment is not necessary except in cases of chronic telogen effluvium, but unfortunately, options are limited (38, 39). While topical minoxidil has mixed results in treating telogen effluvium in adults, its efficacy in children remains unknown (39).

### Anagen Effluvium

Anagen effluvium is rapid hair loss that is rarely seen in the healthy pediatric population. It usually occurs within days or weeks after an inciting event resulting in profound alopecia affecting 90% of the scalp, corresponding to the number of hairs typically in the anagen phase (31, 40). Anagen effluvium should be suspected in a patient with a history of radiotherapy; antineoplastic agents (doxorubicin or vincristine) and other medications, including colchicine; toxic chemical exposure (mercury, boron, and thallium); and severe malnutrition (31, 40, 41). These insults acutely disrupt mitotic activity, thus the proliferating cells of the hair bulbs are preferentially targeted, while quiescent stem cells within the follicle remain unaffected (41). Thus, breakages occur at the fragile proximal hair shaft. Dystrophic anagen hairs will often have tapered fractures of hair shafts (31, 41). Once the insult is removed, most patients experience normal hair regrowth with a delay of 1–3 months; however, permanent alopecia may occur depending on the underlying cause (41). It is important to note that pediatric patients treated with myeloablative conditioning with busulfan have been reported to have permanent diffuse alopecia as a potential side effect. In one multicenter study of 240 childhood leukemia patients, 66 received busulfan and 174 received total body irradiation (TBI) as conditioning. In the busulfan group, 25.8% reported alopecia as a long-term side effect of treatment >10 years out from their stem cell transplant, whereas only 2.9% reported this in the TBI-treated group (42). Use of scalp hypothermia (scalp temperature <24°C), scalp compression, and topical minoxidil can reduce hair loss in some adult patients undergoing chemotherapy, but the techniques differed widely in these studies, and the benefit in children remains unclear (41, 43, 44).

### Loose Anagen Syndrome (LAS)

Loose anagen syndrome is a relatively infrequent cause of hair loss, characterized by poor anchoring of anagen hairs to the follicle, resulting in frequent painless hair loss with gentle pulling (31, 45). Patients are born with sparse but normal hair, and generally present between 2 and 7 years of age with unruly hair that is slow growing. Light microscopy and trichogram reveal predominantly loose anagen hairs (>50%), misshapen anagen roots with absent external root sheaths, and ruffled cuticles (46). Trichoscopy findings include rectangular black granular structures, solitary yellow dots, and >90% of follicular units with single hairs (47). LAS is considered autosomal dominant with incomplete penetrance, and a keratin mutation is responsible for several familial cases (46, 48, 49). LAS may also be associated with heritable developmental conditions, such as Noonan syndrome (50, 51). Due to abnormal premature keratinization in the inner root sheath, hair and the inner root sheath are poorly adherent in LAS. Most cases require no treatment as hair spontaneously returns to normal within a few years, but avoiding hair products and styling, and gentle handling may prevent further hair loss (31, 48, 51). Topical minoxidil may benefit LAS patients, particularly those with severe symptoms (46, 49).

### Short Anagen Syndrome (SAS)

Short anagen syndrome is a rare cause of perceived hair loss in children, due to a shortened growth phase of the hair cycle. This is typically a sporadic congenital condition that can be inherited in an autosomal dominant fashion in some cases. Patients present with normal hair density but with very short hairs that never need to be cut, similar to LAS. However, a gentle hair pull in patients with SAS will reveal that the short hairs are shed in the telogen phase in contrast to being shed in the anagen phase in LAS. This condition is benign, and the majority of patients will improve by puberty (52, 53).

## Traumatic Hair Loss

### Trichotillomania

**Figure 5:**
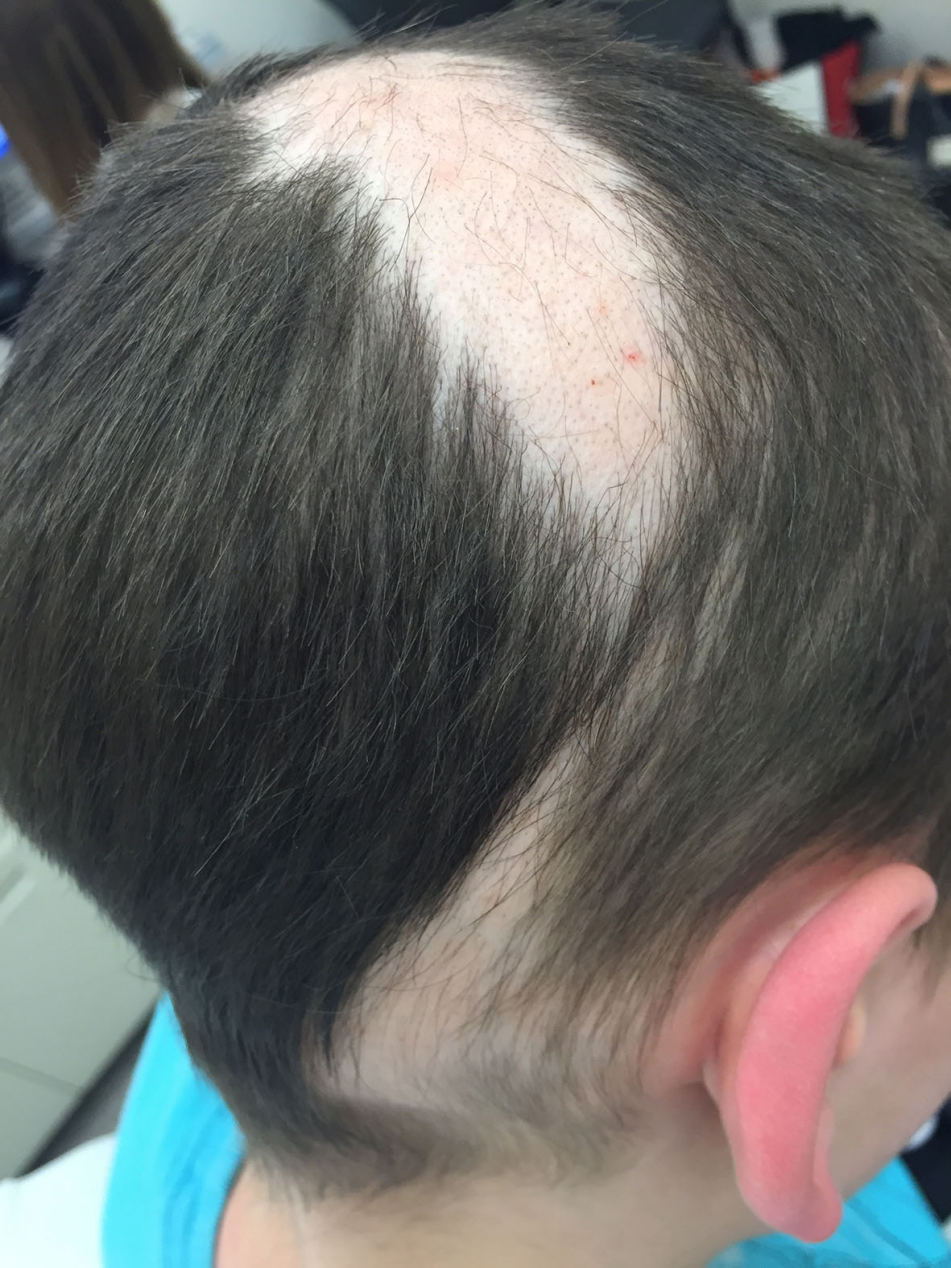
Patchy hair loss characterized by irregular borders and hairs in various stages of regrowth in a child with trichotillomania.

Trichotillomania is a not infrequently seen impulse control disorder characterized by repetitive, compulsive, and self-induced hair pulling from the scalp, eyebrows, and/or other parts of the body (19, 31). Although trichotillomania can occur in children under 6 years old, peak incidence is age 9–13 years with a strong female predominance (54, 55). Clinical presentation includes multiple non-scarring and focal patches of hair loss with irregular borders (Figure 5), little to no scale or excoriation, and presence of broken hairs of different lengths, often contralateral to the side of the body with the dominant hand (19, 56). Hair loss in trichotillomania frequently spares the occiput, as these hairs are more painful to pull out. Trichobezoars can be identified in patients who eat their pulled hairs, leading to gastrointestinal symptoms, including nausea and vomiting, abdominal pain, and bowel perforation (57). In trichotillomania, hair density is normal with a negative pull test; however, presence of flame hairs (proximal hair residue that remains after pulling anagen hairs), the “v-sign” (two hairs rising from the same follicular opening and broken at same level), and tulip hairs (short hairs with darker tulip-shaped ends) on trichoscopy are highly characteristic (58, 59). The main differential diagnosis of trichotillomania is AA. Distinguishing features that are more suggestive of trichotillomania include sparing of the lower occiput scalp, no discrete patches of complete hair loss, a geometric-shaped patch with hairs of all different lengths sometimes with associated evidence of scalp trauma (small pinpoint hemorrhagic spots on scalp). This is in contrast to a patch of hair loss that spontaneously regrows in patients with AA where the hairs typically are all the same length.

Trichotillomania is classified under obsessive–compulsive and related disorders in DSM-V. While its causes are unknown, higher rates of obsessive–compulsive disorder and psychiatric conditions are found in first-degree relatives (56, 60, 61). Management varies across age of presentation. Trichotillomania in preschool-aged children is considered a habit disorder similar to thumb sucking and has a benign course generally disappearing by school age. This can be managed conservatively with gentle reminders. In preadolescents and teenagers, trichotillomania often presents with other psychiatric comorbidities, and patients may benefit from a psychiatric evaluation if willing (19, 55, 56). In children and adolescents, behavioral therapy—positive reinforcement, habit reversal training, and self-monitoring—are effective when working with a skilled therapist (62, 63). Pharmacological interventions are limited. Studies showed mixed results for selective serotonin reuptake inhibitors and tricyclic antidepressants in adults, and data regarding their efficacy in children are lacking (55). While *N*-acetylcysteine is a promising agent that reduces symptoms in adults, no benefit was seen in children (64, 65).

### Traction Alopecia

**Figure 6:**
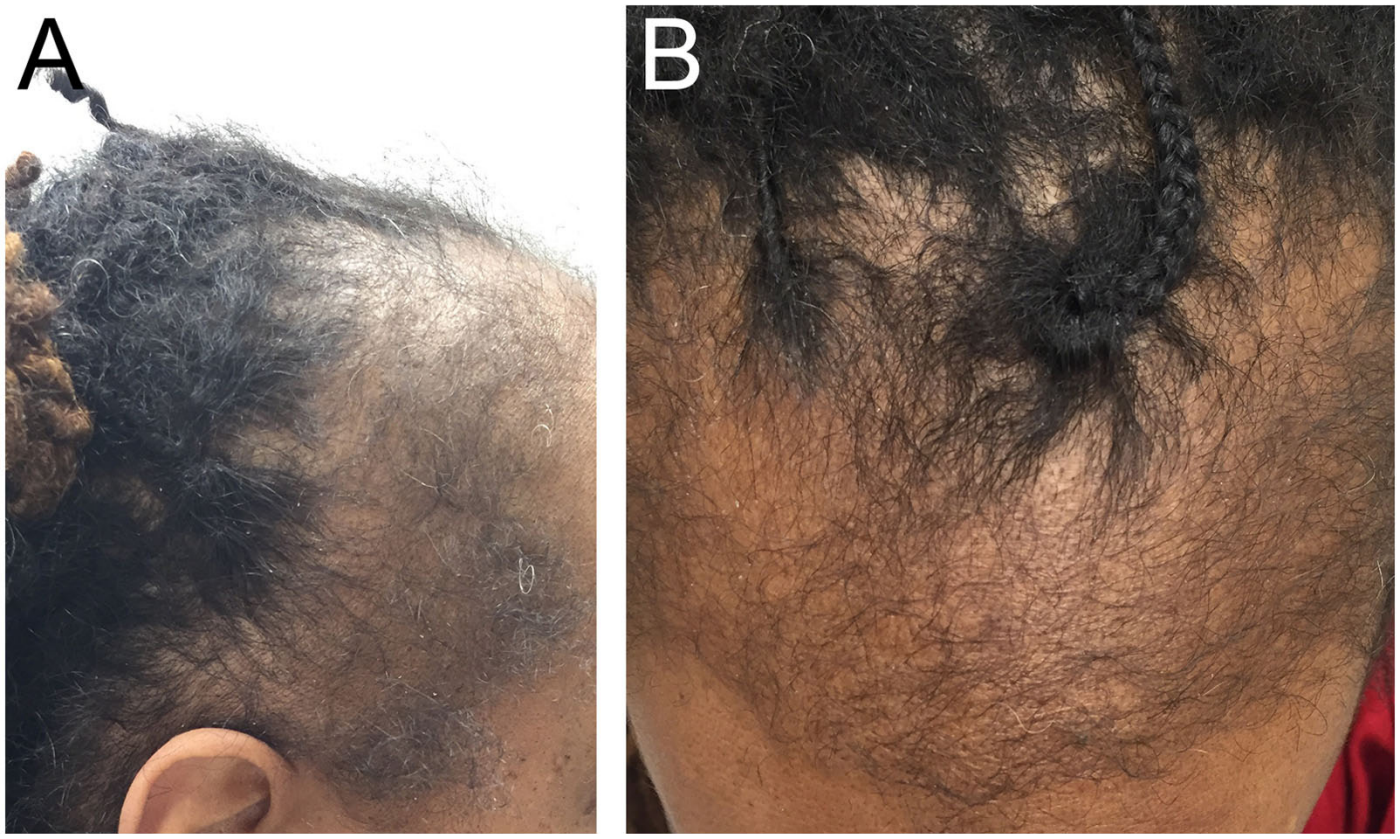
Fringe sign **(A)** and tenting **(B)** are clinical findings suggestive of traction alopecia.

Traction alopecia is a common pattern of traumatic hair loss caused by constant tensile forces exerted on scalp hair. Clinical presentation follows a predictable natural history, with early development of perifollicular erythema, pustules, scaling, and broken hairs and late sequelae of follicular scarring and permanent alopecia. In children, marginal hair loss along the temporal hairline is commonly observed, although non-marginal occipital and frontomarginal patterns can also occur depending on hairstyle (66). Fringe sign is the retention of hairs along the frontal or temporal rim (Figure 6A). Highly suggestive of excessive tension, tenting of hair follicles refers to the raising of skin on the scalp when hair is pulled extremely tightly (Figure 6B). Both are important exam findings for diagnosing traction alopecia (67, 68). The applied trauma is unintentional and often secondary to cultural and social practices given known associations with grooming accessories and particular hairstyles (68, 69). High-risk hairstyling practices include tights buns, ponytails, braids, cornrows, or dreadlocks; application of weaves, braids, or hair extensions to relaxed hair; and any hairstyles that cause pain, crusting, tenting, or pimples (68). Treatment involves adopting grooming practices that minimize traction, and antibiotics to address folliculitis if present (31). With early diagnosis and treatment, regrowth can occur within months; however, chronic traction alopecia may be irreversible despite discontinuation of traction.

### Central Centrifugal Cicatricial Alopecia (CCCA)

Central centrifugal cicatricial alopecia is a form of scarring alopecia that starts with patchy hair loss at the vertex and gradually spreads centrifugally. Patients may complain of tender or pruritic bumps in this location of the scalp. Long thought to be secondary to damage from chemical relaxers and hair dyes, CCCA disproportionately affects middle-aged women of African descent and was previously rarely reported in children (70, 71). However, a recent pediatric case series suggests a familial mode of inheritance with earlier disease onset and greater penetrance in individuals of successive generations. A case series of six children with CCCA reported that the majority (83%) were of African descent, had no history of chemical treatment of hair, and had a positive family history of CCCA (72, 73). Although still a rare cause of hair loss in children, a low threshold of suspicion should be maintained for young African American patients presenting with vertex hair loss and symptoms. To the best of our knowledge, CCCA has not been reported in children of non-African descent. Early treatment with topical or intralesional steroids can decrease inflammation to slow or halt disease progression. This highlights the importance of recognizing CCCA in children with a positive family history to control progressive scarring alopecia.

### Discoid Lupus Erythematosus (DLE)

Discoid lupus erythematosus is very rare in children. It is an inflammatory autoimmune disease that most commonly leads to erythematous, scaly patches on the head and neck that can spread to involve other areas of skin in some cases. The inflammation can lead to atrophy and scarring of the skin and permanent hair loss. Lesions may initially mimic tinea capitis or bacterial infections given the confluent erythema, but laboratory investigations will be negative and there will be a lack of response to anti-infectious agents. The diagnosis is typically made by scalp biopsy revealing basal vacuolization, increased dermal mucin, and a dermal, perivascular, and periadnexal lymphocytic infiltrate. Although there is a female predominance in adult DLE, there is no sex predilection in children. Antinuclear antibodies are uncommon in children (26%), as opposed to 43% positivity in adults; however, progression to systemic lupus erythematosus (SLE) is more common in children with 26% over a 3-year period developing SLE in contrast to only 5% of adults (74). One retrospective study of 40 children with DLE reported the greatest risk of progression to SLE was in the first year after DLE diagnosis. The majority of patients met SLE diagnostic criteria with mucocutaneous disease, cytopenia, and positive antibodies (75). Therefore, children with DLE should be very closely monitored for SLE development. Other goals of management involve controlling activity of disease to prevent further scarring and dyspigmentation. Treatments include potent topical steroids, hydroxychloroquine (4–6 mg/kg/day), and intralesional or systemic corticosteroids. There appears to be some genetic predilection, with DLE patients often reporting a family history of rheumatologic conditions.

### Lichen Planopilaris (LPP)

Lichen planopilaris is an exceedingly rare cause of scarring alopecia in the pediatric population. The cause of LPP is unknown and seems to be sporadic. LPP in adults typically occurs in Caucasian, postmenopausal females. In contrast, LPP in children has no sex predilection and has been reported to occur more frequently in patients of East Indian and African descent. Patients present with scarring alopecia with perifollicular erythema and scales on exam, in contrast to the more confluent erythema and scaling seen in DLE. About half of LPP cases are asymptomatic and could be confused with the more common AA as a result. The clinical features of atrophy and perifollicular scale and erythema are strongly suggestive of LPP (76).

## Aplasia Cutis Congenita (ACC)

**Figure 7:**
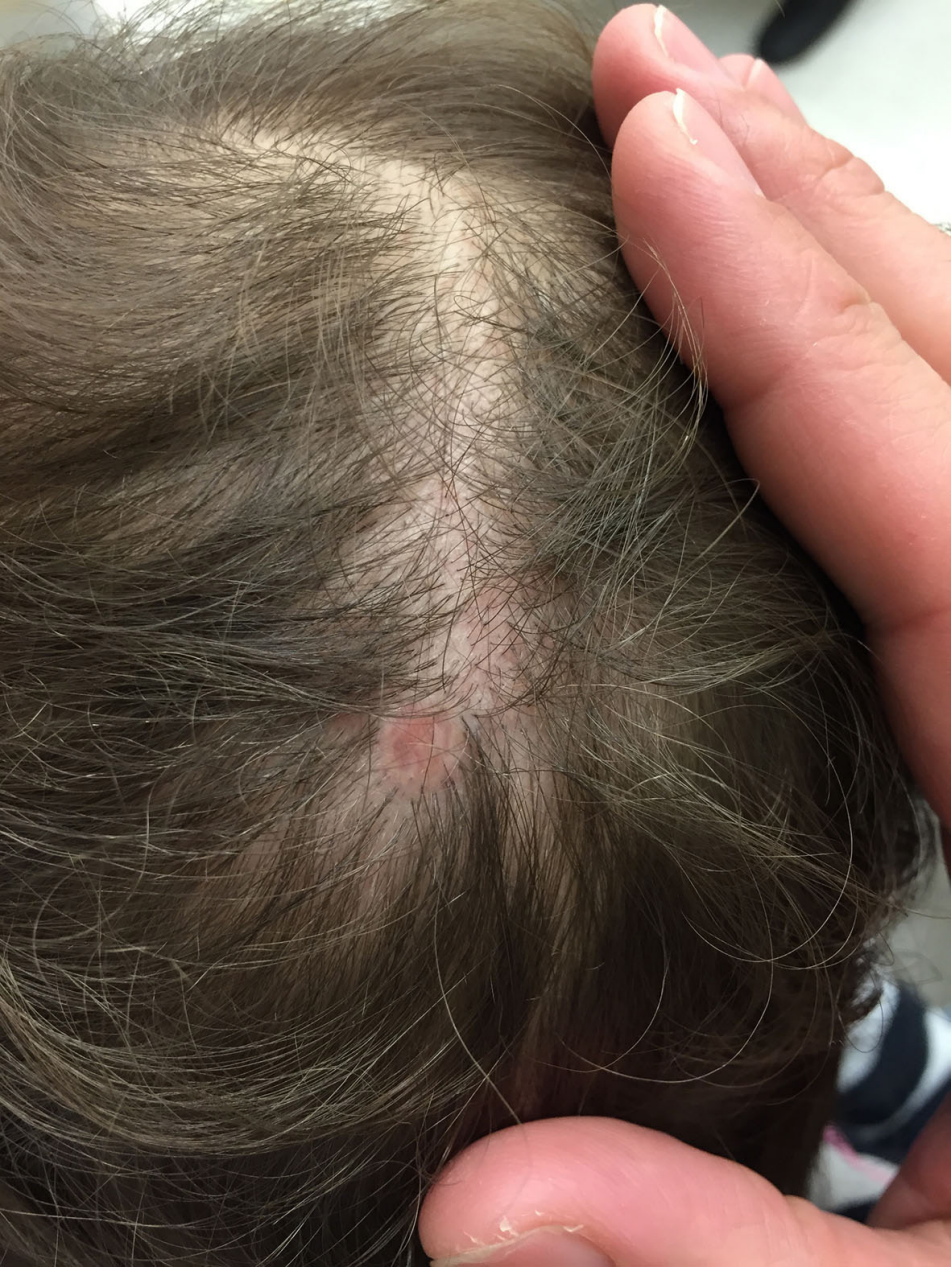
A single, subcentimeter aplasia cutis congenita lesion on the vertex of the scalp.

Aplasia Cutis Congenita (ACC) is a relatively infrequent heterogenous group of diseases characterized by focal absence of skin with accompanying scarring alopecia at birth. Clinical subtypes are characterized by location and pattern of skin absence, presence of associated malformations (e.g., trisomy 13 or 4p-syndrome, cleft lip and palate), and mode of inheritance (77). Although truncal and limb involvement has been described, most patients present with a single midline scalp lesion (Figure 7) (78). Typical lesions are well circumscribed and may be ulcerated or covered by a tense epidermal membrane. However, unlike other neonatal blistering disorders, ACC involves both epidermis and dermis. Proposed etiologies for ACC include infection, vascular malformations, and teratogens affecting aminogenesis or ectodermal fusion, but no unifying theory has been identified (77, 79, 80). Most lesions spontaneously reepithelialize over months, leaving behind a hypertrophic or atrophic scar. Thus, supportive wound care is recommended unless the lesion is greater than 3 cm, in which case split-thickness skin grafting may be indicated (81).

## Other Congenital Alopecia

### Transient Neonatal Hair Loss (TNHL)

Transient neonatal hair loss encompasses the very common phenomenon of neonatal occipital alopecia and development of bald patches in other locations. Healthy babies have been observed to develop alopecic oval patches, most notably in the occiput, during the first 2 months of life. Friction was the presumed cause of TNHL, with the occiput particularly affected due to supine sleeping positions. However, recent retrospective studies found no association between TNHL and sleeping positions. TNHL has a benign course with no accompanying symptoms, and the hair spontaneously regrows (82, 83).

### Congenital Triangular Alopecia (CTA)

Congenital triangular alopecia, also known as temporal triangular alopecia or Brauer nevus, is a non-progressive, non-scarring infrequent cause of alopecia that primarily affects frontotemporal regions. Lesions are triangular or lancet shaped, with complete hair loss of the affected patch except a few vellus hairs. Most lesions are unilateral, predominantly left-sided, but can be bilateral in approximately 7.5–20% of cases (84, 85). A small subset of CTA is associated with known syndromes, including Down syndrome and phakomatosis pigmentovascularis. Although CTA can manifest at birth, most cases are reported after age 2 when the affected patch becomes more noticeable as children undergo vellus to terminal hair transition. Vellus hairs are a highly sensitive but not specific marker for CTA (86). Trichoscopy can demonstrate other features such as miniaturized terminal hair follicles, hair length diversity, and the absence of features characteristic for other types of alopecia, notably AA (85). CTA remains unchanged throughout adult life. Surgical intervention can produce satisfactory cosmetic results, and efficacy of topical minoxidil has been described in a 1-year-old patient (87–90).

### Congenital Atrichia and Hypotrichosis

Congenital atrichia and hypotrichosis comprise a group of rare congenital disorders that present with hair loss or reduction in hair volume (31). Caused by autosomal recessive mutations in the human hairless gene, atrichia congenita presents with either complete hair loss shortly after birth or permanent absence of scalp hair due to follicular agenesis or programmed follicular destruction and papular lesions on the face, scalp, and extremities (31, 91–93). Vitamin-D-dependent rickets is an important differential diagnosis to exclude (93, 94). Atrichia congenita is often mistaken for AU until patients fail to respond to steroid therapy (95). Congenital hypotrichosis is less severe than atrichia congenita with patients presenting after 2 years with diffusely thin hair and variation in quality and quantity of hair (31). Congenital hypotrichosis may be associated with other features, including epilepsy, chromosomal abnormalities, inborn errors of metabolism, and ocular, ectodermal, or skeletal abnormalities. If any associations are identified, workup for a syndromic cause should be performed and genetic counseling may be appropriate depending on the diagnosis (96). For many of these conditions, there is currently no effective treatment.

## Other Patterned Alopecia

### Androgenetic Alopecia (AGA)

Androgenetic Alopecia (AGA) is characterized by relatively common non-scarring hair loss in androgen-dependent regions of the scalp, particularly the vertex and temporoparietal areas, with distinct and well-described male patterns (31, 97, 98). The most frequent cause of alopecia in adolescents, AGA presents gradually after puberty when enough testosterone is converted into dihydrotestosterone and is not generally observed in prepubertal patients without abnormal androgen levels (31, 99, 100). Trichoscopy reveals increased number of thin and vellus hairs, hair shaft thickness diversity, perifollicular pigmentation, and variable number of yellow dots. Biopsy commonly demonstrates perifollicular inflammation (100, 101). A strong genetic predisposition is observed, but no causative gene has been identified, although the androgen receptor gene has been posited (102, 103). Topical minoxidil can prevent progression of AGA in adolescents (99, 100). Finasteride with or without minoxidil is equally well-tolerated and increased hair density in a small cohort of adolescent boys (100). Recent studies have also demonstrated efficacy of low-level laser therapy for adult patients of both genders with AGA (104–106).

### Female Pattern Hair Loss (FPHL)

Female Pattern Hair Loss (FPHL) is a commonly seen non-scarring alopecia distinct from male AGA, presenting with diffuse central thinning beginning anytime after menarche or adrenarche (100, 107). Frontotemporal recession or vertex loss, a more “male pattern” baldness, is less common (107). Trichoscopy shows increased yellow dots, pilosebaceous units with only one hair, and perifollicular hyperpigmentation (108). Family histories for FPHL are often not straightforward, and androgens may not explain all cases of FPHL as many female patients demonstrate little to no biochemical or clinical evidence of increased androgens (107, 109). Signs of hyperandrogenism such as hirsutism and menstrual disturbances should prompt evaluation for polycystic ovarian syndrome, congenital adrenal hyperplasia, and other androgen-producing conditions (110, 111). Topical minoxidil is similarly well tolerated in adolescent girls and prevents progression of FPHL (100). In cases of hyperandrogenism, antiandrogen or 5-alpha reductase inhibitors, such as spironolactone, finasteride, and cyproterone acetate, can be used, though the efficacy of these treatments in children remains unclear (107, 112).

## Hair Shaft Disorders

Hair shaft disorders are acquired or congenital conditions characterized by defects in the hair structure with or without increased breakage. Patients often present with an inability for appropriate hair elongation, abnormal hair texture, or abnormal hair appearance. To help distinguish between the hair shaft disorders, a tug test can be performed to assess for hair fragility (113). Moreover, light microscopy and trichoscopy can identify features specific to different hair shaft disorders (31, 114). Acquired hair shaft disorders such as trichorrhexis nodosa or bubble hair are common and can occur after damage from hair styling, chemicals from hair dyes or permanents, or mechanical trauma from brushing (113). Avoidance of these practices that can be traumatic to the hair shaft will often lead to improvement.

In contrast, congenital hair shaft disorders are very rare and have been associated with many genetic factors, including keratin genes (monilethrix), a transcription factor involved in nucleotide excision repair (trichothiodystrophy), a serine protease inhibitor (trichorrhexis invaginata), genes involved in the urea cycle (trichorrhexis nodosa), and plakoglobin and desmoplakin genes (pili torti, or Woolly hair syndrome) (31, 115). No effective treatments are available for most congenital hair shaft disorders. Some congenital hair shaft disorders such as pili torti may improve over time, while oral retinoids and topical minoxidil improves clinical symptoms of monilethrix (116–121). A comprehensive, algorithmic approach to diagnosing hair shaft disorders has been previously written to address these much less common causes for hair loss and may be used if a hair shaft disorder is suspected (113).

## Conclusion

Alopecia is a relatively frequent presenting problem in the pediatrician’s office and can be due to diverse congenital or acquired conditions. Assessment based on the patient’s age, acute or chronic timeline, and scalp examination can provide extensive clinical insight into the etiology of alopecia. Often a referral to a pediatric dermatologist is helpful, as trichoscopy, light microscopy, and biopsy may not be available in the pediatrician’s office. Physicians can offer psychosocial support with all hair loss complaints. Some alopecia conditions respond to therapeutic interventions, while others resolve over time, especially after removal of the insult. Still others remain permanent without effective therapies. Nonetheless, timely identification of the hair disorders will lead to effective counseling of patients and families about the specific etiologies, prognoses, and available treatment options.

## Author Contributions

LX and KL performed literature review, designed the diagnostic algorithm, drafted the initial manuscript, reviewed and revised the manuscript, and approved the final manuscript as submitted. MS conceptualized and designed the study, drafted the initial manuscript, reviewed and revised the manuscript, and approved the final manuscript as submitted. All the authors approved the final manuscript as submitted and agreed to be accountable for all aspects of the work.

## Conflict of Interest Statement

The authors declare that the research was conducted in the absence of any commercial or financial relationships that could be construed as a potential conflict of interest.

## Abbreviations

AA, alopecia areata; AGA, androgenetic alopecia; ACC, aplasia cutis congenita; CCCA, central centrifugal cicatricial alopecia; CTA, congenital triangular alopecia; DLE, discoid lupus erythematous; FPHL, female pattern hair loss; LAS, loose anagen syndrome; LPP, lichen planopilaris; TNHL, transient neonatal hair loss.

## References

[1] Wolff K Johnson RA Saavedra AP Fitzpatrick’s Color Atlas and Synopsis of Clinical Dermatology (7th Edition). New York: McGraw-Hill Professional Publishing (2009). Available from: https://accessmedicine.mhmedical.com/book.aspx?bookID=1700

[2] Janniger CK Bryngil JM. Hair in infancy and childhood. Cutis (1993) 51(5):336–8. 8513685

[3] Paus R Cotsarelis G The biology of hair follicles. N Engl J Med (1999) 341(7):491–7. 10.1056/NEJM199908123410706 10441606

[4] Inui S. Trichoscopy for common hair loss diseases: algorithmic method for diagnosis. J Dermatol (2011) 38(1):71–5. 10.1111/j.1346-8138.2010.01119.x 21175759

[5] Rudnicka L Rakowska A Kerzeja M Olszewska M. Hair shafts in trichoscopy: clues for diagnosis of hair and scalp diseases. Dermatol Clin (2013) 31(4):695–708, x. 10.1016/j.det.2013.06.007 24075554

[6] Lacarrubba F Micali G Tosti A. Scalp dermoscopy or trichoscopy. Curr Probl Dermatol (2015) 47:21–32. 10.1159/000369402 26370641

[7] Rakowska A Maj M Zadurska M Czuwara J Warszawik-Henzel O Olszewska M Trichoscopy of focal alopecia in children – new trichoscopic findings: hair bulbs arranged radially along hair-bearing margins in aplasia cutis congenita. Skin Appendage Disord (2016) 2(1–2):1–6. 10.1159/000445721 27843914 PMC5096236

[8] Lencastre A Tosti A Role of trichoscopy in children’s scalp and hair disorders. Pediatr Dermatol (2013) 30(6):674–82. 10.1111/pde.12173 23937326

[9] Jackson AJ Price VH. How to diagnose hair loss. Dermatol Clin (2013) 31(1):21–8. 10.1016/j.det.2012.08.007 23159173

[10] Dhurat R Saraogi P. Hair evaluation methods: merits and demerits. Int J Trichology (2009) 1(2):108–19. 10.4103/0974-7753.58553 20927232 PMC2938572

[11] McDonald KA Shelley AJ Colantonio S Beecker J Hair pull test: evidence-based update and revision of guidelines. J Am Acad Dermatol (2017) 76(3):472–7. 10.1016/j.jaad.2016.10.002 28010890

[12] Harries MJ Trueb RM Tosti A Messenger AG Chaudhry I Whiting DA How not to get scar(r)ed: pointers to the correct diagnosis in patients with suspected primary cicatricial alopecia. Br J Dermatol (2009) 160(3):482–501. 10.1111/j.1365-2133.2008.09008.x 19183169

[13] Abdel-Rahman SM Farrand N Schuenemann E Stering TK Preuett B Magie R The prevalence of infections with *Trichophyton tonsurans* in schoolchildren: the CAPITIS study. Pediatrics (2010) 125(5):966–73. 10.1542/peds.2009-2522 20403937

[14] Mirmirani P Tucker L-Y. Epidemiologic trends in pediatric tinea capitis: a population-based study from Kaiser Permanente Northern California. J Am Acad Dermatol (2013) 69(6):916–21. 10.1016/j.jaad.2013.08.031 24094452

[15] Harrison S Sinclair R. Optimal management of hair loss (alopecia) in children. Am J Clin Dermatol (2003) 4(11):757–70. 10.2165/00128071-200304110-00004 14572298

[16] Zaraa I Hawilo A Aounallah A Trojjet S El Euch D Mokni M Inflammatory tinea capitis: a 12-year study and a review of the literature. Mycoses (2013) 56(2):110–6. 10.1111/j.1439-0507.2012.02219.x 22757767

[17] Moriarty B Hay R Morris-Jones R The diagnosis and management of tinea. BMJ (2012) 345:e4380 10.1136/bmj.e4380 22782730

[18] Trovato MJ Schwartz RA Janniger CK Tinea capitis: current concepts in clinical practice. Cutis (2006) 77(2):93–9. 16570671

[19] Castelo-Soccio L. Diagnosis and management of alopecia in children. Pediatr Clin North Am (2014) 61(2):427–42. 10.1016/j.pcl.2013.12.002 24636654

[20] Safavi K Prevalence of alopecia areata in the First National Health and Nutrition Examination Survey. Arch Dermatol (1992) 128(5):702 10.1001/archderm.128.8.1130 1575541

[21] Muller SA Winkelmann RK Alopecia areata. An evaluation of 736 patients. Arch Dermatol (1963) 88:290–7. 10.1001/archderm.1963.01590210048007 14043621

[22] Sharma VK Kumar B Dawn G. A clinical study of childhood alopecia areata in Chandigarh, India. Pediatr Dermatol (1996) 13(5):372–7. 10.1111/j.1525-1470.1996.tb00703.x 8893235

[23] Cooper GS Bynum MLK Somers EC. Recent insights in the epidemiology of autoimmune diseases: improved prevalence estimates and understanding of clustering of diseases. J Autoimmun (2009) 33(3–4):197–207. 10.1016/j.jaut.2009.09.008 19819109 PMC2783422

[24] Petukhova L Duvic M Hordinsky M Norris D Price V Shimomura Y Genome-wide association study in alopecia areata implicates both innate and adaptive immunity. Nature (2010) 466(7302):113–7. 10.1038/nature09114 20596022 PMC2921172

[25] Alkhalifah A Alsantali A Wang E McElwee KJ Shapiro J. Alopecia areata update: part I. Clinical picture, histopathology, and pathogenesis. J Am Acad Dermatol (2010) 62(2):177–88, quiz 189–90. 10.1016/j.jaad.2009.10.032 20115945

[26] Mukherjee N Burkhart CN Morrell DS Treatment of alopecia areata in children. Pediatr Ann (2009) 38(7):388–95. 10.3928/00904481-20090511-07 19685659

[27] Tosti A Guidetti MS Bardazzi F Misciali C. Long-term results of topical immunotherapy in children with alopecia totalis or alopecia universalis. J Am Acad Dermatol (1996) 35(2 Pt 1):199–201. 10.1016/S0190-9622(96)90323-0 8708020

[28] Tan E Tay Y-K Giam Y-C A clinical study of childhood alopecia areata in Singapore. Pediatr Dermatol (2002) 19(4):298–301. 10.1046/j.1525-1470.2002.00088.x 12220271

[29] Hull SM Pepall L Cunliffe WJ. Alopecia areata in children: response to treatment with diphencyprone. Br J Dermatol (1991) 125(2):164–8. 10.1111/j.1365-2133.1991.tb06064.x 1911299

[30] Craiglow BG Liu LY King BA. Tofacitinib for the treatment of alopecia areata and variants in adolescents. J Am Acad Dermatol (2017) 76(1):29–32. 10.1016/j.jaad.2016.09.006 27816292

[31] Alves R Grimalt R Hair loss in children. Curr Probl Dermatol (2015) 47:55–66. 10.1159/000369405 26370644

[32] Headington JT. Telogen effluvium. New concepts and review. Arch Dermatol (1993) 129(3):356–63. 10.1001/archderm.1993.01680240096017 8447677

[33] Harrison S Sinclair R. Telogen effluvium. Clin Exp Dermatol (2002) 27(5):389–385. 10.1046/j.1365-2230.2002.01080.x 12190639

[34] Tuccori M Pisani C Bachini L Pardini M Mantarro S Antonioli L Telogen effluvium following bivalent human papillomavirus vaccine administration: a report of two cases. Dermatology (2012) 224(3):212–4. 10.1159/000337412 22584489

[35] Sinclair R. Chronic telogen effluvium: a study of 5 patients over 7 years. J Am Acad Dermatol (2005) 52(2 Suppl 1):12–6. 10.1016/j.jaad.2004.05.040 15692504

[36] Werner B Mulinari-Brenner F. Clinical and histological challenge in the differential diagnosis of diffuse alopecia: female androgenetic alopecia, telogen effluvium and alopecia areata – part I. An Bras Dermatol (2012) 87(5):742–7. 10.1590/S0365-05962012000600010 23044568

[37] Bedocs LA Bruckner AL. Adolescent hair loss. Curr Opin Pediatr (2008) 20(4):431–5. 10.1097/MOP.0b013e328305e285 18622199

[38] Bardelli A Rebora A Telogen effluvium and minoxidil. J Am Acad Dermatol (1989) 21(3 Pt 1):572–3. 10.1016/S0190-9622(89)80231-2 2633771

[39] Rebora A Telogen effluvium. Dermatology (1997) 195(3):209–12. 10.1159/000245944 9407163

[40] Tosti A Misciali C Piraccini BM Peluso AM Bardazzi F. Drug-induced hair loss and hair growth. Incidence, management and avoidance. Drug Saf (1994) 10(4):310–7. 10.2165/00002018-199410040-00005 8018303

[41] Kanwar AJ Narang T. Anagen effluvium. Indian J Dermatol Venereol Leprol (2013) 79(5):604–12. 10.4103/0378-6323.116728 23974578

[42] Bernard F Auquier P Herrmann I Contet A Poiree M Demeocq F Health status of childhood leukemia survivors who received hematopoietic cell transplantation after BU or TBI: an LEA study. Bone Marrow Transplant (2014) 49(5):709–16. 10.1038/bmt.2014.3 24535128

[43] Grevelman EG Breed WP Prevention of chemotherapy-induced hair loss by scalp cooling. Ann Oncol (2005) 16(3):352–8. 10.1093/annonc/mdi088 15642703

[44] Shin H Jo SJ Kim DH Kwon O Myung S-K. Efficacy of interventions for prevention of chemotherapy-induced alopecia: a systematic review and meta-analysis. Int J Cancer (2015) 136(5):E442–54. 10.1002/ijc.29115 25081068

[45] Price VH Gummer CL. Loose anagen syndrome. J Am Acad Dermatol (1989) 20(2 Pt 1):249–56. 10.1016/S0190-9622(89)70030-X 2915060

[46] Cantatore-Francis JL Orlow SJ. Practical guidelines for evaluation of loose anagen hair syndrome. Arch Dermatol (2009) 145(10):1123–8. 10.1001/archdermatol.2009.220 19841399

[47] Rakowska A Zadurska M Czuwara J Warszawik-Hendzel O Kurzeja M Maj M Trichoscopy findings in loose anagen hair syndrome: rectangular granular structures and solitary yellow dots. J Dermatol Case Rep (2015) 9(1):1–5. 10.3315/jdcr.2015.1193 25932055 PMC4410883

[48] Haskett M Loose anagen syndrome. Australas J Dermatol (1995) 36(1):35–6. 10.1111/j.1440-0960.1995.tb00923.x 7763222

[49] Chapalain V Winter H Langbein L Le Roy J-M Labrèze C Nikolic M Is the loose anagen hair syndrome a keratin disorder? A clinical and molecular study. Arch Dermatol (2002) 138(4):501–6. 10.1001/archderm.138.4.501 11939812

[50] Tosti A Misciali C Borrello P Fanti PA Bardazzi F Patrizi A Loose anagen hair in a child with Noonan’s syndrome. Dermatologica (1991) 182(4):247–9. 10.1159/000247806 1884862

[51] Tosti A Piraccini BM Loose anagen hair syndrome and loose anagen hair. Arch Dermatol (2002) 138(4):521–2. 10.1001/archderm.138.4.521 11939815

[52] Barraud-Klenovsek MM Trüeb RM. Congenital hypotrichosis due to short anagen. Br J Dermatol (2000) 143(3):612–7. 10.1111/j.1365-2133.2000.03720.x 10971339

[53] Herskovitz I de Sousa ICVD Simon J Tosti A. Short anagen hair syndrome. Int J Trichology (2013) 5(1):45–6. 10.4103/0974-7753.114711 23960400 PMC3746230

[54] Walsh KH McDougle CJ. Trichotillomania. Presentation, etiology, diagnosis and therapy. Am J Clin Dermatol (2001) 2(5):327–33. 10.2165/00128071-200102050-00007 11721651

[55] Sah DE Koo J Price VH. Trichotillomania. Dermatol Ther (2008) 21(1):13–21. 10.1111/j.1529-8019.2008.00165.x 18318881

[56] Papadopoulos AJ Janniger CK Chodynicki MP Schwartz RA Trichotillomania. Int J Dermatol (2003) 42(5):330–4. 10.1046/j.1365-4362.2003.01147.x 12755966

[57] Gorter RR Kneepkens CMF Mattens ECJL Aronson DC Heij HA. Management of trichobezoar: case report and literature review. Pediatr Surg Int (2010) 26(5):457–63. 10.1007/s00383-010-2570-0 20213124 PMC2856853

[58] Rakowska A Slowinska M Olszewska M Rudnicka L. New trichoscopy findings in trichotillomania: flame hairs, V-sign, hook hairs, hair powder, tulip hairs. Acta Derm Venereol (2014) 94(3):303–6. 10.2340/00015555-1674 24096547

[59] Tosti A Gray J. Assessment of hair and scalp disorders. J Investig Dermatol Symp Proc (2007) 12(2):23–7. 10.1038/sj.jidsymp.5650051 18004293

[60] King RA Scahill L Vitulano LA Schwab-Stone M Tercyak KP Riddle MA. Childhood trichotillomania: clinical phenomenology, comorbidity, and family genetics. J Am Acad Child Adolesc Psychiatry (1995) 34(11):1451–9. 10.1097/00004583-199511000-00011 8543512

[61] Lenane MC Swedo SE Rapoport JL Leonard H Sceery W Guroff JJ. Rates of obsessive compulsive disorder in first degree relatives of patients with trichotillomania: a research note. J Child Psychol Psychiatry (1992) 33(5):925–33. 10.1111/j.1469-7610.1992.tb01966.x 1634595

[62] Tolin DF Franklin ME Diefenbach GJ Anderson E Meunier SA. Pediatric trichotillomania: descriptive psychopathology and an open trial of cognitive behavioral therapy. Cogn Behav Ther (2007) 36(3):129–44. 10.1080/16506070701223230 17852170

[63] Franklin ME Edson AL Ledley DA Cahill SP. Behavior therapy for pediatric trichotillomania: a randomized controlled trial. J Am Acad Child Adolesc Psychiatry (2011) 50(8):763–71. 10.1016/j.jaac.2011.05.009 21784296 PMC3143367

[64] Grant JE Odlaug BL Kim SW. N-acetylcysteine, a glutamate modulator, in the treatment of trichotillomania: a double-blind, placebo-controlled study. Arch Gen Psychiatry (2009) 66(7):756–63. 10.1001/archgenpsychiatry.2009.60 19581567

[65] Bloch MH Panza KE Grant JE Pittenger C Leckman JF. N-acetylcysteine in the treatment of pediatric trichotillomania: a randomized, double-blind, placebo-controlled add-on trial. J Am Acad Child Adolesc Psychiatry (2013) 52(3):231–40. 10.1016/j.jaac.2012.12.020 23452680 PMC3745012

[66] Hantash BM Schwartz RA. Traction alopecia in children. Cutis (2003) 71(1):18–20. 12553624

[67] Samrao A Price VH Zedek D Mirmirani P The “Fringe Sign” – a useful clinical finding in traction alopecia of the marginal hair line. Dermatol Online J (2011) 17(11):1 10.4172/2155-9554.1000117 22136857

[68] Haskin A Aguh C. All hairstyles are not created equal: what the dermatologist needs to know about black hairstyling practices and the risk of traction alopecia (TA). J Am Acad Dermatol (2016) 75(3):606–11. 10.1016/j.jaad.2016.02.1162 27114262

[69] Steinberg S Ezers IA Alopecia in women. Can Fam Physician (1970) 16(4):64–6. PMC2281685 20468499

[70] Gathers RC Lim HW. Central centrifugal cicatricial alopecia: past, present, and future. J Am Acad Dermatol (2009) 60(4):660–8. 10.1016/j.jaad.2008.09.066 19293013

[71] Shah SK Alexis AF. Central centrifugal cicatricial alopecia: retrospective chart review. J Cutan Med Surg (2010) 14(5):212–22. 10.2310/7750.2010.09055 20868618

[72] Eginli AN Dlova NC McMichael A Central centrifugal cicatricial alopecia in children: a case series and review of the literature. Pediatr Dermatol (2017) 34(2):133–7. 10.1111/pde.13046 27981623

[73] Dlova NC Jordaan FH Sarig O Sprecher E Autosomal dominant inheritance of central centrifugal cicatricial alopecia in black South Africans. J Am Acad Dermatol (2014) 70(4):679–82.e1. 10.1016/j.jaad.2013.11.035 24480456

[74] Miettunen PMH Bruecks A Remington T. Dramatic response of scarring scalp discoid lupus erythematosus (DLE) to intravenous methylprednisolone, oral corticosteroids, and hydroxychloroquine in a 5-year-old child. Pediatr Dermatol (2009) 26(3):338–41. 10.1111/j.1525-1470.2009.00916.x 19706100

[75] Arkin LM Ansell L Rademaker A Curran ML Miller ML Wagner A The natural history of pediatric-onset discoid lupus erythematosus. J Am Acad Dermatol (2015) 72(4):628–33. 10.1016/j.jaad.2014.12.028 25648823

[76] Christensen KN Lehman JS Tollefson MM. Pediatric lichen planopilaris: clinicopathologic study of four new cases and a review of the literature. Pediatr Dermatol (2015) 32(5):621–7. 10.1111/pde.12624 26058419

[77] Mesrati H Amouri M Chaaben H Masmoudi A Boudaya S Turki H Aplasia cutis congenita: report of 22 cases. Int J Dermatol (2015) 54(12):1370–5. 10.1111/ijd.12707 26016611

[78] Fagan L-L Harris PA Coran AG Cywes R. Sporadic aplasia cutis congenita. Pediatr Surg Int (2002) 18(5–6):545–7. 10.1007/s00383-002-0812-5 12415408

[79] Drolet B Prendiville J Golden J Enjolras O Esterly NB “Membranous aplasia cutis” with hair collars. Congenital absence of skin or neuroectodermal defect? Arch Dermatol (1995) 131(12):1427–31. 10.1001/archderm.131.12.1427 7492133

[80] Drolet BA Baselga E Gosain AK Levy ML Esterly NB. Preauricular skin defects. A consequence of a persistent ectodermal groove. Arch Dermatol (1997) 133(12):1551–4. 10.1001/archderm.1997.03890480071010 9420540

[81] Liu Y Qiu L Fu Y Tian X Yuan X Xiao J Large defects in aplasia cutis congenita treated by large-sized thin split-thickness skin grafting: long-term follow-up of 18 patients. Int J Dermatol (2015) 54(6):710–4. 10.1111/ijd.12773 26010404

[82] Cutrone M Grimalt R Transient neonatal hair loss: a common transient neonatal dermatosis. Eur J Pediatr (2005) 164(10):630–2. 10.1007/s00431-005-1707-y 16010567

[83] Kim MS Na CH Choi H Shin BS. Prevalence and factors associated with neonatal occipital alopecia: a retrospective study. Ann Dermatol (2011) 23(3):288–92. 10.5021/ad.2011.23.3.288 21909197 PMC3162256

[84] Yamazaki M Irisawa R Tsuboi R. Temporal triangular alopecia and a review of 52 past cases. J Dermatol (2010) 37(4):360–2. 10.1111/j.1346-8138.2010.00817.x 20507407

[85] Fernández-Crehuet P Vaño-Galván S Martorell-Calatayud A Arias-Santiago S Grimalt R Camacho-Martínez FM Clinical and trichoscopic characteristics of temporal triangular alopecia: a multicenter study. J Am Acad Dermatol (2016) 75(3):634–7. 10.1016/j.jaad.2016.04.053 27543220

[86] Inui S Nakajima T Itami S Temporal triangular alopecia: trichoscopic diagnosis. J Dermatol (2012) 39(6):572–4. 10.1111/j.1346-8138.2011.01348.x 21906133

[87] Wu WY Otberg N Kang H Zanet L Shapiro J Successful treatment of temporal triangular alopecia by hair restoration surgery using follicular unit transplantation. Dermatol Surg (2009) 35(8):1307–10. 10.1111/j.1524-4725.2009.01233.x 19496794

[88] Unger R Alsufyani MA. Bilateral temporal triangular alopecia associated with phakomatosis pigmentovascularis type IV successfully treated with follicular unit transplantation. Case Rep Dermatol Med (2011) 2011:129541. 10.1155/2011/129541 23198168 PMC3504251

[89] Chung J Sim JH Gye J Namkoong S Hong SP Kim MH Successful hair transplantation for treatment of acquired temporal triangular alopecia. Dermatol Surg (2012) 38(8):1404–6. 10.1111/j.1524-4725.2012.02442.x 22672444

[90] Bang C-Y Byun J-W Kang M-J Yang B-H Song H-J Shin J Successful treatment of temporal triangular alopecia with topical minoxidil. Ann Dermatol (2013) 25(3):387–8. 10.5021/ad.2013.25.3.387 24003292 PMC3756214

[91] Ahmad W Zlotogorski A Panteleyev AA Lam H Ahmad M Faiyaz ul Haque M Genomic organization of the human hairless gene (HR) and identification of a mutation underlying congenital atrichia in an Arab Palestinian family. Genomics (1999) 56(2):141–8. 10.1006/geno.1998.5699 10051399

[92] Zlotogorski A Ahmad W Christiano AM. Congenital atrichia in five Arab Palestinian families resulting from a deletion mutation in the human hairless gene. Hum Genet (1998) 103(4):400–4. 10.1007/s004390050840 9856480

[93] Yip L Horev L Sinclair R Zlotogorski A. Atrichia with papular lesions: a report of three novel human hairless gene mutations and a revision of diagnostic criteria. Acta Derm Venereol (2008) 88(4):346–9. 10.2340/00015555-0466 18709303

[94] Miller J Djabali K Chen T Liu Y Ioffreda M Lyle S Atrichia caused by mutations in the vitamin D receptor gene is a phenocopy of generalized atrichia caused by mutations in the hairless gene. J Invest Dermatol (2001) 117(3):612–7. 10.1046/j.0022-202x.2001.01438.x 11564167

[95] Zlotogorski A Panteleyev AA Aita VM Christiano AM. Clinical and molecular diagnostic criteria of congenital atrichia with papular lesions. J Invest Dermatol (2002) 118(5):887–90. 10.1046/j.1523-1747.2001.01767.x 11982770

[96] De Berker D Congenital hypotrichosis. Int J Dermatol (1999) 38(Suppl 1):25–33. 10.1046/j.1365-4362.1999.00005.x 10369537

[97] Ludwig E. Classification of the types of androgenetic alopecia (common baldness) occurring in the female sex. Br J Dermatol (1977) 97(3):247–54. 10.1111/j.1365-2133.1977.tb15179.x 921894

[98] Ellis JA Sinclair R Harrap SB. Androgenetic alopecia: pathogenesis and potential for therapy. Expert Rev Mol Med (2002) 4(22):1–11. 10.1017/S1462399402005112 14585162

[99] Price VH. Androgenetic alopecia in adolescents. Cutis (2003) 71(2):115–21. 12635889

[100] Gonzalez ME Cantatore-Francis J Orlow SJ. Androgenetic alopecia in the paediatric population: a retrospective review of 57 patients. Br J Dermatol (2010) 163(2):378–85. 10.1111/j.1365-2133.2010.09777.x 20346026

[101] Kibar M Aktan S Bilgin M. Scalp dermatoscopic findings in androgenetic alopecia and their relations with disease severity. Ann Dermatol (2014) 26(4):478–84. 10.5021/ad.2014.26.4.478 25143677 PMC4135103

[102] Hillmer AM Hanneken S Ritzmann S Becker T Freudenberg J Brockschmidt FF Genetic variation in the human androgen receptor gene is the major determinant of common early-onset androgenetic alopecia. Am J Hum Genet (2005) 77(1):140–8. 10.1086/431425 15902657 PMC1226186

[103] Ellis JA Stebbing M Harrap SB. Polymorphism of the androgen receptor gene is associated with male pattern baldness. J Invest Dermatol (2001) 116(3):452–5. 10.1046/j.1523-1747.2001.01261.x 11231320

[104] Kim H Choi JW Kim JY Shin JW Lee SJ Huh C-H Low-level light therapy for androgenetic alopecia: a 24-week, randomized, double-blind, sham device-controlled multicenter trial. Dermatol Surg (2013) 39(8):1177–83. 10.1111/dsu.12200 23551662

[105] Jimenez JJ Wikramanayake TC Bergfeld W Hordinsky M Hickman JG Hamblin MR Efficacy and safety of a low-level laser device in the treatment of male and female pattern hair loss: a multicenter, randomized, sham device-controlled, double-blind study. Am J Clin Dermatol (2014) 15(2):115–27. 10.1007/s40257-013-0060-6 24474647 PMC3986893

[106] Afifi L Maranda EL Zarei M Delcanto GM Falto-Aizpurua L Kluijfhout WP Low-level laser therapy as a treatment for androgenetic alopecia. Lasers Surg Med (2017) 49(1):27–39. 10.1002/lsm.22512 27114071

[107] Olsen EA Messenger AG Shapiro J Bergfeld WF Hordinsky MK Roberts JL Evaluation and treatment of male and female pattern hair loss. J Am Acad Dermatol (2005) 52(2):301–11. 10.1016/j.jaad.2004.04.008 15692478

[108] Rakowska A Slowinska M Kowalska-Oledzka E Olszewska M Rudnicka L. Dermoscopy in female androgenic alopecia: method standardization and diagnostic criteria. Int J Trichology (2009) 1(2):123–30. 10.4103/0974-7753.58555 20927234 PMC2938574

[109] Norwood OT Lehr B. Female androgenetic alopecia: a separate entity. Dermatol Surg (2000) 26(7):679–82. 10.1046/j.1524-4725.2000.99310.x 10886278

[110] Lin-Su K Nimkarn S New MI. Congenital adrenal hyperplasia in adolescents: diagnosis and management. Ann N Y Acad Sci (2008) 1135:95–8. 10.1196/annals.1429.021 18574213

[111] Chang RJ. A practical approach to the diagnosis of polycystic ovary syndrome. Am J Obstet Gynecol (2004) 191(3):713–7. 10.1016/j.ajog.2004.04.045 15467530

[112] Yazdabadi A Green J Sinclair R. Successful treatment of female-pattern hair loss with spironolactone in a 9-year-old girl. Australas J Dermatol (2009) 50(2):113–4. 10.1111/j.1440-0960.2009.00517.x 19397563

[113] Mirmirani P Huang KP Price VH. A practical, algorithmic approach to diagnosing hair shaft disorders. Int J Dermatol (2011) 50(1):1–12. 10.1111/j.1365-4632.2010.04768.x 21182495

[114] Miteva M Tosti A. Dermatoscopy of hair shaft disorders. J Am Acad Dermatol (2013) 68(3):473–81. 10.1016/j.jaad.2012.06.041 22940404

[115] Cheng AS Bayliss SJ The genetics of hair shaft disorders. J Am Acad Dermatol (2008) 59(1):1–22; quiz 23–6. 10.1016/j.jaad.2008.04.002 18571596

[116] Hernández-Pérez E Letter: tretinoin therapy for monilethrix. Arch Dermatol (1974) 109(4):575–6. 10.1001/archderm.109.4.575 4819126

[117] Tamayo L Monilethrix treated with the oral retinoid Ro 10-9359 (Tigason). Clin Exp Dermatol (1983) 8(4):393–6. 10.1111/j.1365-2230.1983.tb01799.x 6627730

[118] De Berker D Dawber RP. Monilethrix treated with oral retinoids. Clin Exp Dermatol (1991) 16(3):226–8. 10.1111/j.1365-2230.1991.tb00356.x 1934581

[119] Saxena U Ramesh V Misra RS Topical minoxidil in monilethrix. Dermatologica (1991) 182(4):252–3. 10.1159/000247808 1884864

[120] Karincaoglu Y Coskun BK Seyhan ME Bayram N. Monilethrix: improvement with acitretin. Am J Clin Dermatol (2005) 6(6):407–10. 10.2165/00128071-200506060-00008 16343029

[121] Rossi A Iorio A Scali E Fortuna MC Mari E Palese E Monilethrix treated with minoxidil. Int J Immunopathol Pharmacol (2011) 24(1):239–42. 10.1177/039463201102400129 21496408

